# Optimizing tacrolimus dosing in Hispanic renal transplant patients: insights from real-world data

**DOI:** 10.3389/fphar.2024.1443988

**Published:** 2024-09-19

**Authors:** Athanasios Chamzas, Eglis Tellez, Andrew SyBing, Jogarao V. S. Gobburu, Mathangi Gopalakrishnan

**Affiliations:** ^1^ Center for Translational Medicine, University of Maryland School of Pharmacy, Baltimore, MD, United States; ^2^ Providence St Joseph, Eureka, CA, United States

**Keywords:** tacrolimus, renal transplant, hispanic population, population pharmacokinetic modeling, real world data

## Abstract

**Aim:**

Tacrolimus, an immunosuppressant used to prevent organ rejection in renal transplant patients, exhibits high inter-patient variability, necessitating therapeutic drug monitoring. Early post-transplant tacrolimus exposure in Hispanics is understudied. Although genotypic information is linked to pharmacokinetic differences, its clinical application remains limited. This study aimed to use a real-world data-driven, pharmacokinetic model-based approach for tacrolimus in Hispanics to determine a suitable initial dose and design an optimal dose titration strategy by simulations to achieve plasma trough concentration target levels of 10–12 ng/mL at the earliest.

**Methods:**

Sparse concentration-time data of tacrolimus were obtained from electronic medical records for self-identified Hispanic subjects following renal transplant. Rich pharmacokinetic literature data was leveraged to estimate structural pharmacokinetic model parameters, which were then fixed in the current analysis. Only apparent clearance was estimated with the sparse tacrolimus data and potential covariates were identified. Simulations of various starting doses and different dose titration strategies were then evaluated.

**Results:**

The analysis included 121 renal transplant patients with 2,215 trough tacrolimus concentrations. A two-compartment transit absorption model with allometrically scaled body weight and time-varying hematocrit on apparent clearance adequately described the data. The estimated apparent clearance was 13.7 L/h for a typical patient weighing 70 kg and at 30% hematocrit, demonstrating a 40% decrease in clearance compared to other patient populations. Model based simulations indicated the best initial dose for the Hispanic population is 0.1 mg/kg/day. The proposed titration strategy, with three dose adjustments based on trough levels of tacrolimus, increased the proportion of patients within the target range (10–12 ng/mL) more than 2.5-fold and decreased the proportion of patients outside the therapeutic window by 50% after the first week of treatment.

**Conclusion:**

Hispanic renal transplant population showed an estimated 40% decrease of apparent clearance in the typical patient compared to other populations with similar characteristics. The proposed dose adjustment attained the target range rapidly and safely. This study advocates for tailored tacrolimus dosing regimens based on population pharmacokinetics to optimize therapy in Hispanic renal transplant recipients.

## 1 Introduction

Tacrolimus is a potent immunosuppressant drug commonly used in renal transplantation to prevent organ rejection. Because tacrolimus has a narrow therapeutic range, therapeutic drug monitoring is necessary to ensure adequate immunosuppression while avoiding serious adverse effects, including infections, nephrotoxicity, and neurotoxicity (Venkataramanan et al., 1995). Whole-blood trough concentrations are highly correlated with clinical outcomes, and hence used to monitor and adjust the dosage of patients individually (Staatz et al., 2001).

However, despite numerous studies conducted on tacrolimus, optimizing its dose is challenging due to high pharmacokinetic between subject variability (BSV) (5%–93%) and within subjects (Zuo et al., 2013), especially in understudied populations like the Hispanic population, as dosing regimens are based on studies primarily conducted in White populations. Following oral administration, tacrolimus is rapidly absorbed, highly bound to erythrocytes and plasma proteins (Zahir et al., 2001) with an absolute bioavailability ranging between 25%–30% (Venkataramanan et al., 1991; Staatz and Tett, 2004). The bioavailability is mainly influenced by the extensive first pass metabolism (through cytochrome isoenzymes CYP3A4 and CYP3A5) and efflux of the drug back into the intestinal lumen by P-glycoprotein (Benet, 1998; Tuteja et al., 2001). Although genotype has been associated with 1.5 to 2-fold differences in pharmacokinetic parameters (Brooks et al., 2016), and models incorporating genotype have improved the proportion of patients reaching target concentrations (Thervet et al., 2010), the routine collection and use of patients’ genotype in clinical practice remains limited.

Given the high inter-subject variability and the narrow therapeutic target range (10–12 ng/mL) for tacrolimus, characterizing the drug in specific patient populations becomes paramount. One such population that warrants exploration is Hispanics or Latinos, who are 1.3 times more likely to have renal failure compared to White Americans (Race and Kidney Disease, 2016), thereby necessitating a greater need for renal transplantation in the Hispanic population. However, there is a paucity of literature or studies investigating early post-transplant tacrolimus exposure in the Hispanic population, which could potentially inform optimal dosing.

Real-world data from electronic medical records, where essential information is systematically gathered during routine clinical practice, can be harnessed to address these therapeutic questions. The use of electronic medical records provides a cost-effective alternative to resource-intensive clinical trials in patients. Although genotype has been linked to explaining the pharmacokinetic variability of tacrolimus (Brooks et al., 2016), patients’ genotype are not widely collected or used in clinical practice today, limiting their practical applications for dose adjustments. However, a population pharmacokinetic model-based approach that leverages easily retrievable patient-specific information from electronic health records (EHR) during patient admission could assist clinicians in making objective decisions regarding dose adjustments.

Therefore, the study aimed to (i) develop a population pharmacokinetic (popPK) model that adequately describes tacrolimus pharmacokinetics in Hispanic renal transplant patients using EHR data, (ii) analyze the impact of subject-specific factors, (iii) determine a suitable initial tacrolimus dose for the Hispanic population, and (iv) design an optimal dose titration strategy to maximize the percentage of patients achieving the target range of 10–12 ng/mL at the earliest.

## 2 Materials and methods

### 2.1 Patient selection

This retrospective study complies with the legal requirements and the ethical standard of the Declaration of Helsinki and was approved by the Hospital Ethics Committee (Exempt Status 20–076). Data were collected retrospectively from electronic medical records of patients who received an allograft renal transplant at St. Joseph Hospital, Orange, CA between the years 2008 and 2020. Individuals who self-identified as Hispanics were included in the study. Patients were excluded if they experienced delayed graft function necessitating the cessation of tacrolimus treatment to commence dialysis, if they had incomplete dosing records or missing covariate information.

Covariates collected for each subject were age at transplant, gender, body weight, body mass index (BMI), serum creatinine, creatinine clearance, number of transplants, length of stay, infections after transplant, use of calcium channel blockers, graft status, delayed graft function, donor type, albumin, aspartate transaminase, alanine transaminase, hematocrit, and total bilirubin.

### 2.2 Dosage regimen

All patients received an immunosuppressive regimen consisting of tacrolimus orally twice daily, induction with thymoglobulin, started with 500 mg of Methylprednisoloneon operation day and gradually decreased to Prednisone 5 mg by time of discharge with further reductions to 10 mg during clinic visit, 1 g oral twice daily mycophenolate mofetil or 500 mg twice daily if the patient experienced side effects. Tacrolimus was introduced when renal function was improving with adequate urinary output, and a serum creatinine ≤3 mg/dL, initial dose (0.05–0.15 mg/kg/day), which was adjusted based on observed morning trough level concentrations. Following hospital protocol, the target trough concentration range was 10–12 ng/mL after transplant for the first 3 months, 8–10 ng/mL for 4–12 months, and 5–8 ng/mL thereafter.

### 2.3 Blood sample collection

Blood samples were collected before the morning dose of the patient measuring the trough concentration of tacrolimus. The samples were analyzed using liquid chromatography with tandem mass spectrometry (LC/MS) following lab protocol (Christians et al., 2000).

The patient data were retrieved for the first 30 days of their treatment, retrospectively using the patient database TeleResults Presidio. The drug levels were collected following patients’ clinical visits. In the first week, trough levels were available almost daily or every other day. In the subsequent weeks, the trough levels were available once or twice a week. Given the retrospective nature of the data, some dosing events were missing. Dosing events that were missing but with records of trough concentrations, were imputed using the next dose record available. Additionally, concentrations that were reported as below the Lower Limit of Quantification (LLQ) were treated as missing (1.7% of concentration values).

### 2.4 Population pharmacokinetic analysis

The popPK analysis was performed using the non-linear mixed effects modeling approach using the First-Order Conditional Estimation (FOCE) method in Pumas (JuliaPro Version 1.7.2, Pumas Version 2.3.0).

### 2.5 Structural model

Since only trough concentrations were collected, the data was sparse to determine the structural model and estimate the pharmacokinetic parameters. While a plethora of pharmacokinetic models for tacrolimus exists in the literature, the published models vary widely in their structural model specification (i.e., number of compartments, absorption processes), thereby adding complexity to the model selection process. To address this, publicly available rich individual patient data from a bioequivalence trial conducted by Alloway et al. (2017) was leveraged to determine the structural model parameters. As individual doses were not reported, the median dose was selected for all subjects, and a sensitivity analysis was performed to assess the impact of selecting different initial doses on the estimated parameters. For the sensitivity analysis, doses from the interquartile range (0.04–0.08 mg/day) of the Alloway et al. trial were iteratively selected, and each dose was used to estimate PK parameters from the rich data. With all parameters except clearance fixed, the sparse data were then used to evaluate the influence of the selected initial dose on the estimation of this parameter of interest, clearance. Additionally, these PK estimates were cross-referenced with results from similar structural model studies to ensure they aligned with reported ranges.

The bioequivalence trial (Alloway et al., 2017) included 35 renal transplant patients above 18 years old with stable organ function and no evidence of rejection. Median (IQR) age was 52 (39.0–59.0) and 65.7% of the participants were males. After receiving the same dose for 7 days, the individuals reached a steady state, and then 15 samples were collected from each patient in a 12-h window from the time before the morning dose until the next dose. In each study sequence, the reference tacrolimus formulation was administered twice. The individual profiles digitized for this study were from the reference formulation’s first administration.

Considerable variability exists in the literature concerning the compartmental and absorption models for tacrolimus. One-, two-, and three-compartment structural pharmacokinetic (PK) models were evaluated to characterize tacrolimus disposition, and various absorption processes were explored, including first-order, transit, and zero-order absorption.

Random effects on PK parameters for between-subject variabilities were assumed to follow a log-normal distribution with a mean of zero and a variance of ω^2^. For the within-subject variability or unexplained residual error, different error models, including additive, proportional, or combined, were assessed. Residual error was assumed to follow a normal distribution with a mean of zero and a variance of σ^2^. Selection among competing models was carried out using statistical criteria such as changes in the minus twice the log-likelihood (-2LL, ≥3.84; χ^2^, df = 1, α = 0.05), Akaike information criterion (AIC), Bayes information criterion (BIC), and precision of parameter estimates, while also considering biological plausibility.

Once the base model was deemed adequate for the rich tacrolimus data, parameter estimates for absorption and all other structural PK parameters except apparent central clearance were fixed when analyzing the sparse data. The BSV for the above pharmacokinetic parameters were also fixed given the sparse nature of the data and the similarity of the population characteristics with the rich tacrolimus data (renal transplant patients receiving oral tacrolimus BID). For the sparse data, only apparent clearance (CL/F) and its variability was estimated.

### 2.6 Covariate model

After the structural model was determined, the effect of subject-specific covariates on clearance and volume parameters, as appropriate, were evaluated graphically. Only covariates with adequate correlation and physiological importance were included in the model. The impact of both continuous and categorical covariates on pharmacokinetic parameters was assessed using Equation 1 and Equation 2 respectively:

$$\theta_{i}=\theta_{pop}\cdot {\left(\frac{COV}{median\left(COV\right)}\right)}^{\theta_{COV}}$$ (1)

$$\theta_{i}=\theta_{pop}\cdot \left(1+\theta_{COV}\cdot \left(COV\right)\right)$$ (2)

COV is the covariate value and θ_COV_ is the covariate effect. Standard forward selection and backward elimination processes were used to select the covariates. Statistical criteria for the covariate to be included in the final PK model were reduction in the minus twice the log-likelihood (-2LL, 3.84 or greater; χ^2^, df = 1, α = 0.05). Reduction in BSV after the covariate inclusion was also considered.

### 2.7 Final model qualification

The final model qualification was performed using a combination of goodness of fit diagnostics, precision of the parameter estimates, and quantitative predictive checks (QPCs) (Jadhav and Gobburu, 2005). The precision of the final model parameters was determined using asymptotic standard errors and a bootstrap procedure with 500 bootstrap replicates.

For the QPCs, the metric used to evaluate the model was the trough concentration at 72 h, because it represents the time point at which the patient can be assumed to have reached steady state and is important for the first dose adjustment. Given that there were no observations for all patients at exactly 72 h, a window of 66–74 h was used for the QPC analysis. The final population parameter estimates from the model were used to simulate 1,000 replicates of the same dosing timings, covariate information as in the original dataset. The 50th percentile of the trough concentration within this window was obtained and collected according to when the actual observation was made. A histogram of the 50th percentiles was plotted and overlaid with the 50th percentile of the observed data for the same hour window, providing a visual comparison of the model’s predictive performance. The prediction error (%PE) to evaluate the model’s accuracy was calculated with Equation 3:

$$\%PE=\frac{\left(PRED-OBS\right)*100}{OBS}$$ (3)

Where PRED is the predicted median Ctrough at 72 h in each of the 1,000 simulated datasets and OBS is the observed median value Ctrough. The fifth, 50th, and 95th percentiles of %PE were calculated and reported.

### 2.8 Simulations

Using the *post hoc* individual pharmacokinetic parameters from the developed popPK model and preserving the population’s covariate information, multiple scenarios were simulated to evaluate the initial tacrolimus dose and to derive an optimal dose titration strategy. As the simulations used the individual predictions without residual error each titration strategy was simulated once.

The dose adjustments were based on commercially available doses, with a minimum change of 0.5 mg. In line with clinical practice, simulated blood sampling was done at the morning trough dose, and dose changes were implemented for the subsequent evening dose. The maximum single dose administered could not exceed 13 mg, as seen in the observed dataset. Since therapeutic drug monitoring is highly patient- and physician-specific, simulated results were compared to observed concentration data. A range of different starting doses (0.05–0.20 mg/kg/day) and varied dose titration strategies (e.g.,: X% increase or decrease in dose based on tacrolimus levels below or above target respectively) were evaluated based on: the percentage of patients inside target range (10–12 ng/mL) on each day, above safety threshold (15 ng/mL), below efficacy threshold (5 ng/mL), median days to first achieve target range and clinical applicability. Titration strategies were developed to align with clinically relevant approaches, taking into consideration the dosing protocol currently in use at St. Joseph hospital (Orange, CA). The dose proportionality Equation 4 as shown below:

$${Dose}_{new}=\frac{{Dose}_{old}\cdot \;{Concentration}_{measured}}{{Concentration}_{target}}$$ (4)

was employed to calculate the necessary adjustment windows and doses. Multiple concentration thresholds and corresponding dose adjustments were then systematically tested to identify the most effective dose titration strategy.

Tacrolimus’s average half-life is about 12 h (Wallemacq and Verbeeck, 2001) and it takes on average four to five half-lives to achieve steady state after the initial dose. Consequently, assessing the performance of different initial doses becomes clinically relevant on days 2 and 3, as steady-state conditions are approached. In the event of a dose adjustment, blood samples were collected after a 3-day interval to allow the drug to reach steady-state levels. Based on the data, all patients receive MMF (mycophenolate mofetil) and the recommended initial dose is 0.10 mg/kg/day according to the drug’s packet insert (FDA label, 2022).

## 3 Results

**FIGURE 1:**
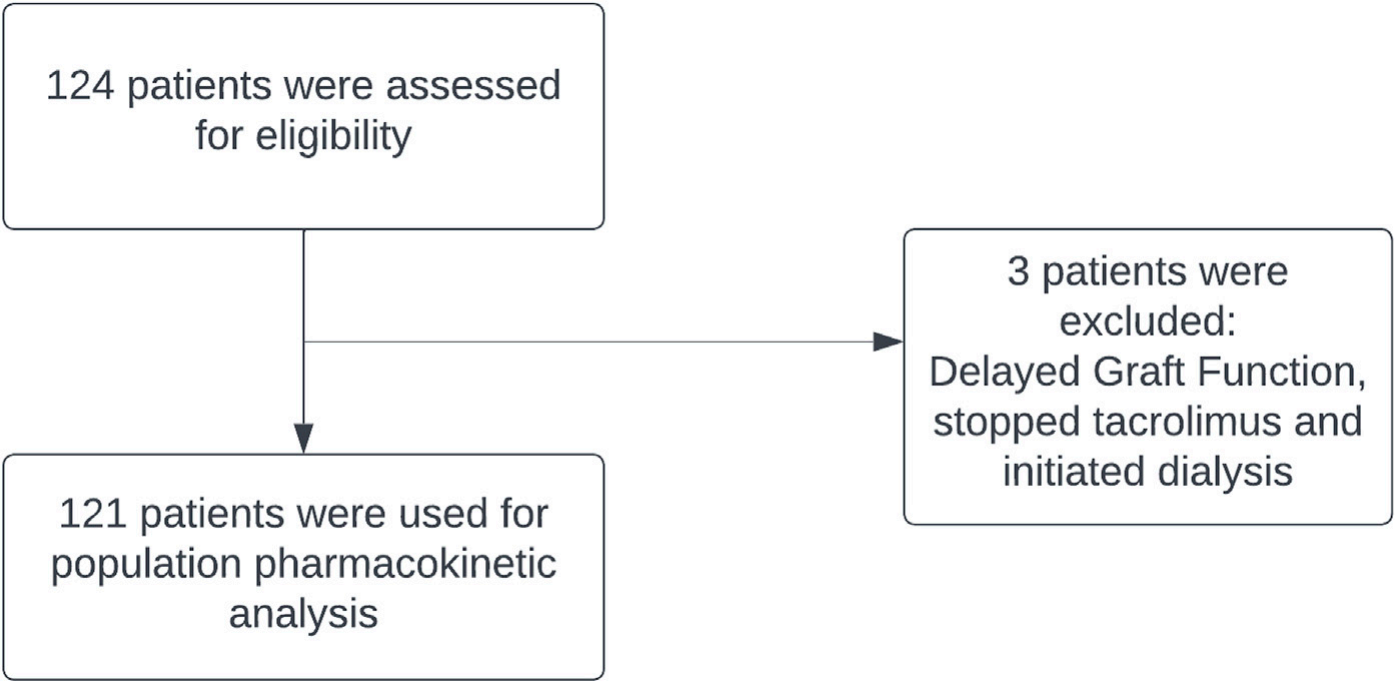
Flowchart. Data preparation flowchart.

Figure 1 shows the screening process of patients according to the eligibility criteria, resulting in the inclusion of 121 patients for analysis from an initial assessment of 124 individuals and 2,215 trough tacrolimus concentrations. Baseline demographics of the population are summarized in Table 1. Average number of trough concentrations per patient was 18, and the initial tacrolimus dose ranged between 0.02 and 0.14 mg/kg/day. Out of 7,570 dosing records, 110 were missing and were imputed based on the next available recorded dose. These imputations were made for patients who lacked dosing and concentration data during their initial days of therapy but had subsequent recorded doses and concentrations.

**TABLE 1:** Baseline Characteristics of Hispanic population receiving renal transplant.

Patient Characteristic (n = 121)	Values
Gender (no. [%]) •Male •Female	74 (61%) 47 (39%)
No. Of Transplants (no. [%]) •First •Second •Third	105 (87) 14 (12) 2 (1.7)
Donor Type (no. [%]) •Deceased •Living	86 (71) 35 (29)
Age (mean, [SD]) (years)	45.7 (12.9)
Serum Creatinine (mean, [SD]) (mg/dL)	4.7 (2.6)
Creatinine Clearance (mean, [SD]) (mL/min)	29.9 (17.5)
Weight (mean, [SD]) (kg)	77.3 (20.1)
BMI (mean, [SD]) (kg/m^2^)	27.6 (5.2)
Albumin (mean, [SD]) (g/dL)	3.6 (0.6)
Aspartate aminotransferase (AST) (mean, [SD]) (units/l)	22.6 (11.6)
Alanine transaminase (ALT) (mean, [SD]) (units/l)	17.3 (12.2)
Total bilirubin (mean, [SD]) (mg/dL)	0.7 (0.4)
Hematocrit (HCT) (mean, [SD]) (%)	30.0 (4.9)

### 3.1 Base model

A two-compartment model with linear elimination and transit absorption process adequately described tacrolimus pharmacokinetics for the rich sampling study. This finding aligns with the results of a similar rich sampling study conducted by Benkali et al. (Benkali et al., 2009). For the transit absorption model, three transit compartments was found to be adequate. Model building steps using the bioequivalence trial (Alloway et al., 2017) data are provided in Supplementary Table S1. Sensitivity analysis results are shown in Supplementary Figure S1.

For the sparse dataset, apparent volume of distribution of the central compartment (Vc/F), apparent volume of distribution of the peripheral compartment (Vp/F), apparent inter-compartmental clearance (Q/F), and transfer rate (Ktr) were fixed to 171 L, 325.6 L, 62.5 L/h, and 4.27 h^-1^ respectively as obtained from the rich data analysis. The estimated apparent CL/F was 13.7 L/h with a BSV of 77.5%. The additive residual error was estimated as 2.62 ng/mL.

### 3.2 Covariate model

Based on exploratory graphical analysis, body weight and hematocrit showed meaningful trends with respect to random effects of CL/F. Inclusion of allometrically scaled weight as a covariate to CL/F, Vc/F, Vp/F, and Q/F decreased the -2LL by 24.2 units. Inclusion of allometrically scaled weight and allometrically scaled hematocrit on clearance decreased -2LL by 100 units.

**FIGURE 2:**
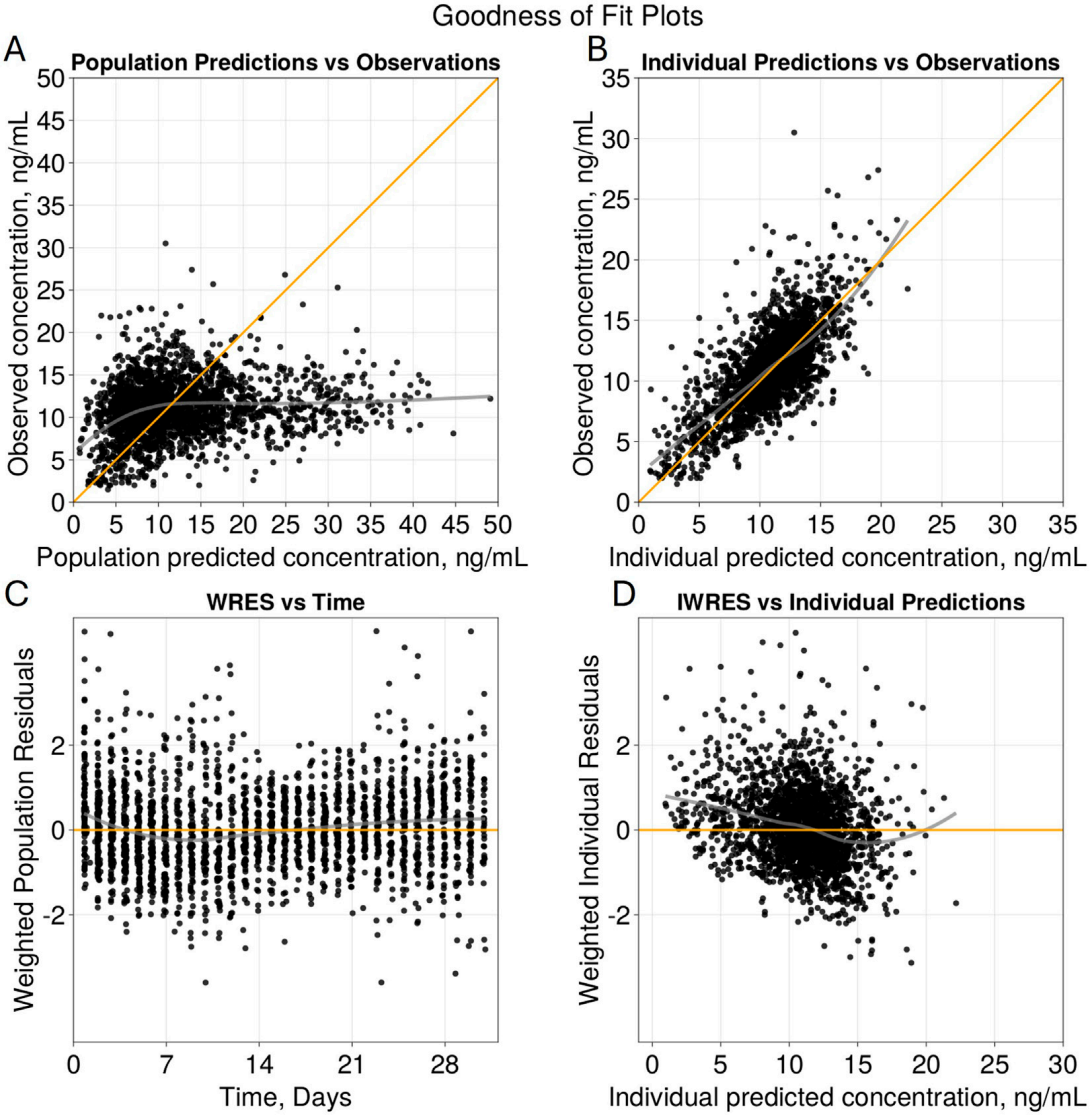
Goodness of Fit Plots for Final Model. Diagnostic plots of the final two compartment transit absorption model **(A)** observed *versus* population predicted tacrolimus concentrations (ng/mL), **(B)** observed *versus* individual predicted tacrolimus concentrations (ng/mL), **(C)** weighted residuals *versus* time (days), and **(D)** weighted residuals *versus* individual predictions (ng/mL). Yellow lines in **(A)** and **(B)** represent line of identity and grey indicates trend line.

**FIGURE 3:**
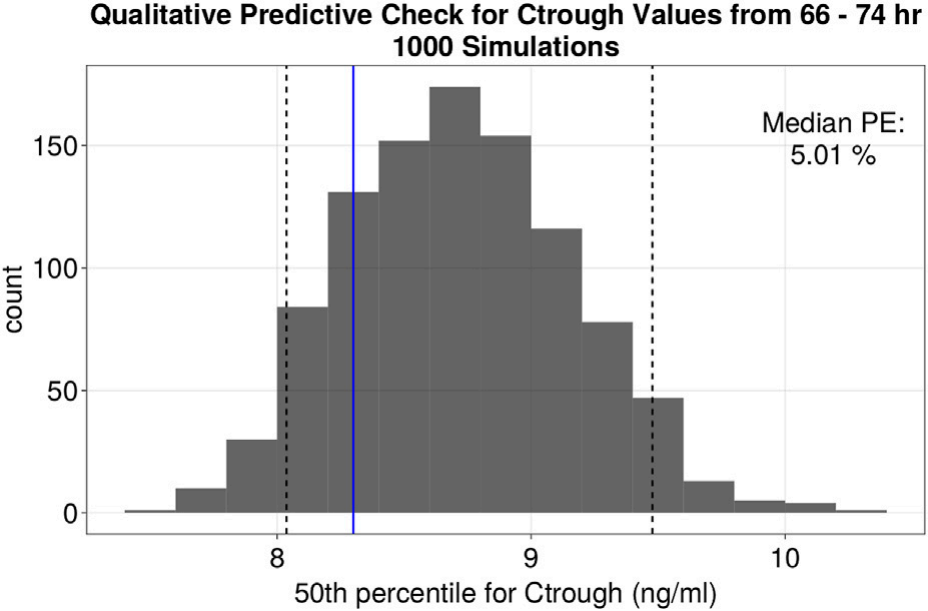
Quantitative predictive check of Ctrough levels at 72 h (66–74 h window). Histogram of the distribution of the median values of Ctrough at 72 h (ng/mL) of 1,000 simulations. Black dotted lines represent the 5th and 95th percentile of the median values from the simulations. The fifth, 50th and 95th percentiles of %PE for Ctrough at 72 h were −3.17, 5.01% and 14.19% respectively.

**FIGURE 4:**
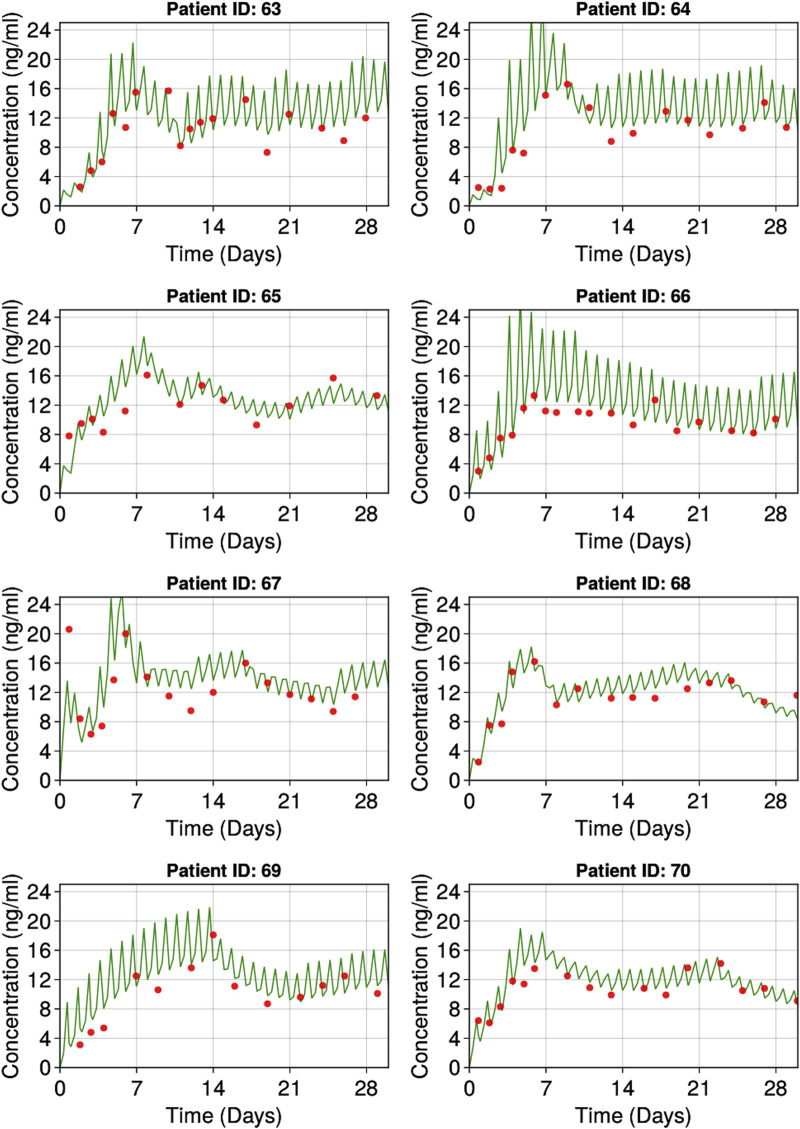
Representative individuals goodness of fit plots. Red dots represent observed individual tacrolimus concentrations. Green lines represent individual predicted tacrolimus concentrations.

The goodness of fit plots for the final model are shown in Figure 2 and the qualitative predictive check for trough concentration at 72 h is shown in Figure 3. Representative individual concentration profiles are shown in Figure 4. Table 2 displays the final PK parameter estimates, along with their precision quantified as the relative standard error (RSE%). The estimated PK parameters, namely, apparent clearance and HCTcov, demonstrate precise estimation with RSE% values consistently below 30% and the 95% CI obtained from 500 bootstrap replicates indicate the robustness of estimation process. The final covariate model Equations 5–9 are listed below:

$$CL=tvcl*{\left(\frac{HT}{HT\_median}\right)}^{-HTcov}*{{\left(\frac{Wt}{70}\right)}^{0.75}*\;e}^{\eta CL}$$ (5)

$$Vc=tvvc*{\left(\frac{Wt}{70}\right)*\;e}^{\eta Vc}$$ (6)

$$Vp=tvvp*{\left(\frac{Wt}{70}\right)*\;e}^{\eta Vp}$$ (7)

$$Q=tvq*{{\left(\frac{Wt}{70}\right)}^{0.75}*\;e}^{\eta Q}$$ (8)

$$Ktr=tvktr*e^{\eta Ktr}$$ (9)

**TABLE 2:** Final population pharmacokinetic model parameter estimates.

Population parameter	Mean estimate of typical value	%RSE of typical value	%BSV	%RSE of BSV	Shrinkage (%)	Bootstrap 95% CI (n = 200)
CL/F, L/hr^a^	13.65	4.32%	77.46	12.24%	4.3	(12.6–14.86)
V_c_/F, L^b^	171 (fixed)	-	118.78 (fixed)	-	-	-
V_p_/F, L^c^	325 (fixed)	-	102.09 (fixed)	-	-	-
Q/F, L/hr^d^	62.46 (fixed)	-	106.19 (fixed)	-	-	-
Ktr, hr^-1^ ^e^	4.27 (fixed)	-	86.44 (fixed)	-	-	-
HCTcov effect on clearance	0.83	8.46%	-		-	(0.6–1.03)
RUV – Additive Error (ng/mL)	2.62	12.85%	-		-	(2.45–2.82)

CL/F: apparent clearance; V_c_/F: apparent volume of distribution of the central compartment; V_p_/F: apparent volume of distribution of peripheral compartment; Q/F: apparent Inter-compartmental clearance; Ktr: transfer rate; HCTcov: hematocrit exponent estimate on clearance; BSV: between subject variability; RUV: residual unexplained variability; %RSE: percent relative standard error; CI: confidence interval.

The (fixed) parameters use the estimated typical values from the rich sampling study.

^a^ CL , tvcl * (HCT/(HCT_median)) ^−HCTcov^ * (Wt/70)0.75 * e^ηCL^

^b^ Vc = tvvc * (Wt/70) * e^ηVc^.

^c^ Vp = tvvp * (Wt/70) * e^ηVp^.

^d^ Q = tvq * (Wt/70)0.75 * e^ηQ^.

^e^ Ktr = tvktr * e^ηKtr^.

### 3.3 Simulations

#### 3.3.1 Initial dose

**FIGURE 5:**
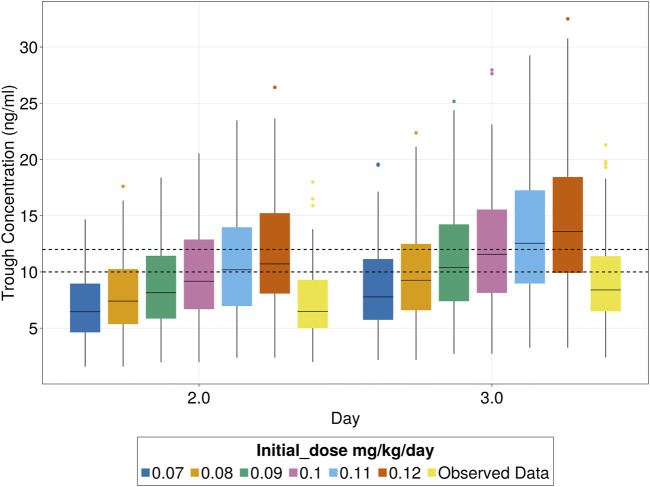
Tacrolimus patients inside target trough concentration range (10–12 ng/mL) for different initial doses.

The simulations indicated that using an initial dose of 0.09 mg/kg/day increased the percentage of patients reaching the target range (10–12 ng/mL) from 15% to 16.4% by day 3, with no increase in patients exceeding the safety limit (10.6% observed vs 10.7% with 0.09 mg/kg/day). Since 0.09 mg/kg/day is nearly equivalent to the current standard dose of 0.1 mg/kg/day, no adjustment to the initial dose is recommended. For subsequent simulations, a dose of 0.1 mg/kg/day was used, which increased the percentage of patients within the target range from 15% to 27% but also elevated the proportion exceeding the safety limit (15 ng/mL) from 10.6% to 18%. This highlights the need for dose titration before reaching steady state for those at risk of exceeding the safety limit. The median days to first achieve the target range decreased from 5 to 4 days. These findings are shown in Figure 5.

#### 3.3.2 Dose adjustment

Various dose titration strategies were comprehensively evaluated to maximize the proportion of subjects reaching the target trough concentrations for tacrolimus and minimize the time to achieve the target. The titration strategy was developed considering the specific needs of Hispanic subjects who may experience decreased clearance and a higher susceptibility to exceeding safety threshold (>15 ng/mL). An initial dose of 0.1 mg/kg/day could cause adverse effects in patients with low clearance levels. To mitigate this risk, patients with concentrations above 9 ng/mL on Day 2 had their dose reduced by 50% to prevent exceeding the safety threshold.

**FIGURE 6:**
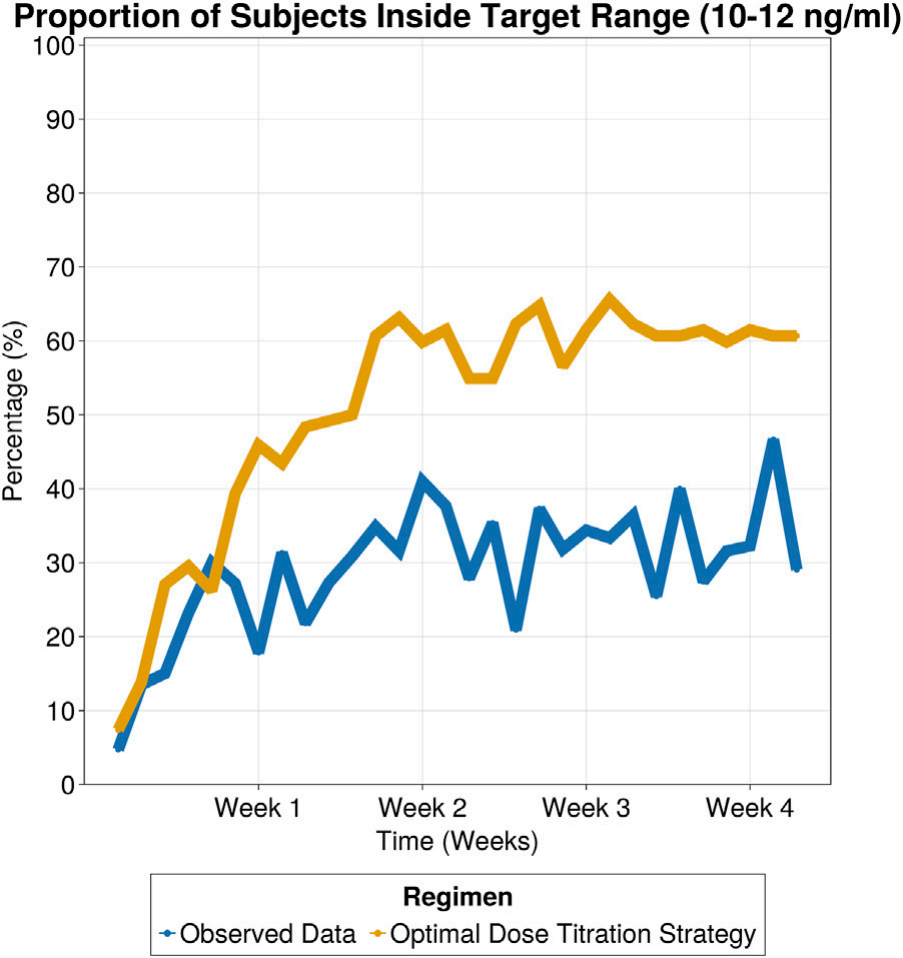
Proportion of subjects inside target range (10–12 ng/mL) for observed data and simulated patients following optimal dose titration strategy.

**FIGURE 7:**
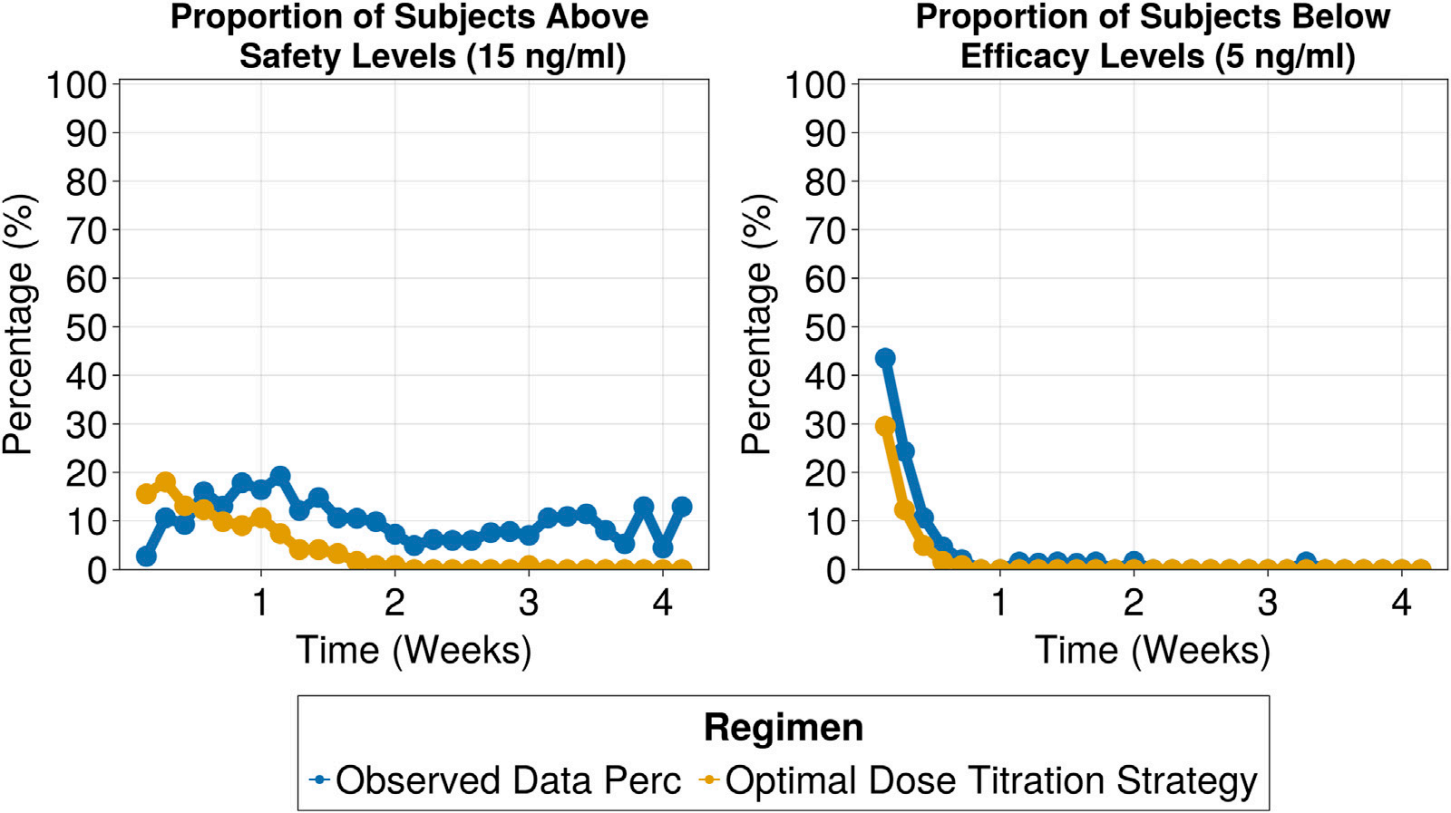
Proportion of patients above safety threshold (above 15 ng/mL) (Left) and proportion of patients below efficacy threshold (below 5 ng/mL) (Right).

The selected titration strategy involved three dose adjustment windows based on tacrolimus trough levels, with corresponding recommended dose adjustments outlined in Table 3. By employing the proposed dose titration strategy, on day 7 the percentage of patients within the target range increased to 46%, compared to 18% in the observed data. This 2.5-fold improvement, which remained consistent throughout the 30-day treatment period (as shown in Figure 6), led to a significant reduction in the proportion of subjects exceeding safety thresholds (Figure 7). On Day 7, only 9% of patients were above safety thresholds, in contrast to the observed data of 17.9%. Furthermore, at 2 weeks of treatment, less than 1% of patients remained above safety threshold compared to 9.8% from the observed data.

**TABLE 3:** Dose titration recommended protocol.

		Trough Concentration Level (ng/mL)	Recommended Titration
Day 2		On day 2 if the trough concentration is above 9	reduce 50%
Day 3 Onward	Below	<7	50% increase
		7–9	1 time 50% increase +25% maintenance
		9–9.5	25% increase
	Above	12.5–13	reduce 25%
		13–15	skip next dose and reduce 25%
		15<	skip next dose and reduce 50%

## 4 Discussion

Despite extensive research on tacrolimus pharmacokinetics, a gap remains in understanding tacrolimus exposure during the early post-transplant period in Hispanic kidney transplant population. This gap is significant due to the drug’s narrow therapeutic window, high variability, and the 1.3-fold higher prevalence of renal failure in Hispanics compared to white Americans (Race and Kidney Disease, 2016). Current dosing guidelines for tacrolimus are primarily based on studies conducted in mainly White populations, potentially leading to suboptimal dosing in other ethnic groups. Significant pharmacokinetic differences warranting dose adjustments for tacrolimus have been observed in other populations to ensure adequate target attainment. For example, African Americans exhibit a 2-fold higher clearance of tacrolimus than Whites, necessitating higher initial dosages (Sanghavi et al., 2017), to achieve comparable drug blood levels. This was attributed to a higher allele prevalence in CYP3A5 enzyme that leads to extensive drug metabolism.

Previous pharmacokinetic studies in the Mexican population (García-Roca et al., 2012; Reséndiz-Galván et al., 2019) have highlighted the need for individualized dosing informed by genotypic variations. However, genotypic information is not routinely collected in clinical practice, limiting the applicability of such personalized approaches. While conducting clinical trials for each population is not feasible, the availability of electronic medical records can help bridge these knowledge gaps. This study aimed to use a real-world data driven, population pharmacokinetic model-based approach to characterize the pharmacokinetics of tacrolimus in the Hispanic population, to optimize dosing regimens to improve target attainment.

Given the sparse tacrolimus concentrations available for the study subjects obtained from the electronic medical records, digitized data from a rich sampling study of tacrolimus was leveraged to derive the structural PK model. Tacrolimus pharmacokinetics was adequately described by a two compartment model with transit absorption similar to the rich sampling study similar to Benkali et al. (Benkali et al., 2009). With the structural PK model established, apparent clearance was estimated using the trough concentrations available from the Hispanic study population. Body weight and hematocrit explained the between subject variability in apparent clearance from 69% to 67%.

Hematocrit was found to be negatively correlated with tacrolimus clearance similar to previous literature reports (Benkali et al., 2009; Brooks et al., 2016). A transplant patient’s hematocrit is typically lower than normal right after surgery. As erythrocytes and plasma proteins increase with time, tacrolimus binds to them, resulting in decreased clearance over time as there is less free drug to be cleared (Campagne et al., 2019). In the current study, it was observed that a typical subject with hematocrit (HCT) of 50% will have a 30% decrease in clearance compared to the normal HCT 30% (Supplementary Table S2).

**FIGURE 8:**
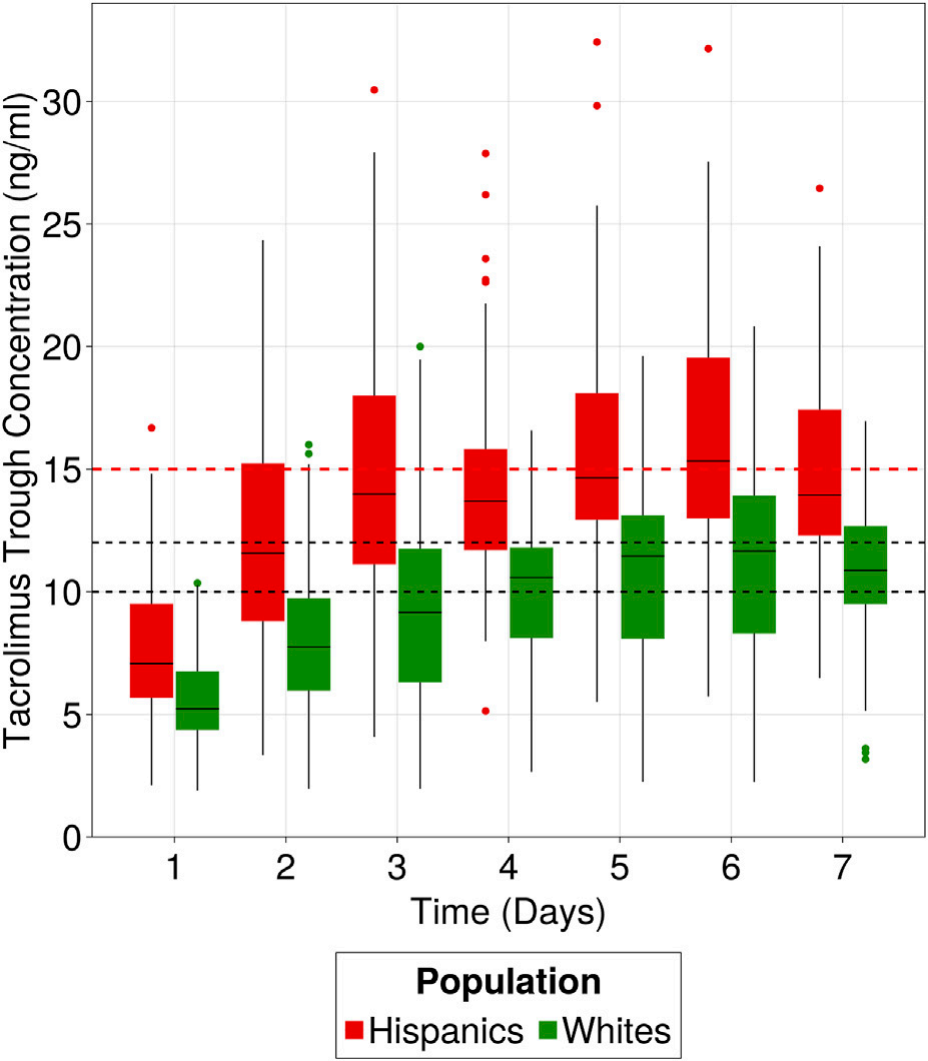
Clinical implication of Hispanic population difference in clearance. Hispanics and Whites receiving 0.1 mg/kg/day as initial dose and following hospital’s dose titration strategy for the first week of treatment. Target range is 10–12 ng/mL and safety level is 15 ng/mL. Data were simulated using the developed popPK model to simulate 100 patients from the Hispanic population and 100 patients using the 23.5 L/h clearance, representative of “White” populations from literature.

An estimated 40% reduction in apparent clearance for the typical Hispanic patient (13.7 L/h) was observed when compared to non-Hispanic populations with similar characteristics acquired from rich data publications consisting of mainly White population (mean apparent clearance value 23.5 L/h with a range [20.5–28](Benkali et al., 2009; Woillard et al., 2011; Bergmann et al., 2014; Han et al., 2014; Størset et al., 2014)). The clinical implications of this difference in apparent clearance are depicted in Figure 8 through simulations (n = 100), comparing Hispanic subjects with an estimated clearance of 13.7 L/h (based on our data) to White subjects with a mean clearance of 23.5 L/h, derived from the above publications, all following hospital’s current dose titration strategy. Notably, the Hispanic population failed to reach the target range (10–12 ng/mL) during the first week of treatment, even surpassing the safety threshold when compared to the White population. Only 14% of simulated Hispanics were inside the target range compared to 38% of Whites after 1 week of treatment.

To validate the finding of decreased clearance, the observed tacrolimus trough concentrations from the current study in the Hispanic population (receiving a median initial dose of 0.06 mg/kg/day) were descriptively compared with the observed concentrations reported by Francke et al. (Francke et al., 2021) (Supplementary Figure S2). In the Francke et al. study, patients were administered a median dose of 0.1 mg/kg/day for 5 consecutive doses until the morning of postoperative day 3, and the population was predominantly Whites (>90%). Despite receiving almost half the dose in Hispanic population, the two populations exhibited similar concentrations on Day 3 (8.3 ng/mL and 8.4 ng/mL), indicating that the Hispanic population has decreased drug clearance. Additionally, on Day 5, the median trough concentration in the Hispanic population was 24% higher than that of the White population.

Similar findings of lower apparent clearance have been reported in Mexican (Reséndiz-Galván et al., 2019) and Native American (Grover et al., 2011) populations. Pharmacogenomics could account for the decreased clearance in the Hispanic population, given that tacrolimus clearance is mediated primarily through CYP3A5. However, evaluating the impact of known mutations in CYP3A5, CYP3A4, and P-glycoprotein which can affect tacrolimus clearance was not feasible, as the information was not available. CYP3A5 is the primary enzyme associated with tacrolimus clearance (Brooks et al., 2016), with CYP3A5*3 being the most common single nucleotide polymorphism (SNP) observed in the Hispanic population in the United States (Claudio-Campos et al., 2015). This SNP is frequently associated with a reduction in functional CYP3A5 enzyme activity or complete loss of protein function. Nevertheless, this observation alone cannot explain the reduced apparent clearance rates, as other populations, including Whites, also predominantly exhibit the CYP3A5*3 variant. Hence, although a clear reason for the 40% lower apparent clearance in the Hispanic population could not be ascertained through this study, other potential reasons such as ethnic distinctions potentially related to diet, a combination of other enzymatic factors, or variations in pathophysiology that may affect bioavailability may be considered. Table 4 provides a summary of clearance estimates available from literature across diverse populations.

**TABLE 4:** Comparative tacrolimus oral clearance estimates from the literature.

References	Population	Number of subjects	CL/F ± SE
-	Hispanics	121	13.7 ± 0.6 L/h
Reséndiz-Galván et al. (2019)	Mexicans	52	12.3 ± 0.98 L/h
Grover et al. (2011)	Native Americans	24	11.1 ± 5.53 L/h
Andrews et al. (2019)	Whites	237	23 ± 0.69 L/h
Benkali et al. (2009)	French	32	28 ± 4 L/h
Woillard et al. (2011)	French	32	20.3 ± 1.3 L/h
Sanghavi et al. (2017)	African Americans	212	54.6 ± 5.46 L/h
Bergmann et al. (2014)	Australian	173	25.5 L/h ± 1.6 L/h

The proposed dose titration strategy specifically tailored for Hispanic renal transplant subjects could objectively inform clinical practice. The dose titration strategy consisted of three dose adjustment windows based on tacrolimus trough levels. This approach increased the percentage of patients within the target range by 2.5-fold after 1 week of treatment compared to the observed data and halved the number of subjects outside the therapeutic window. These improvements were sustained over the 30-day treatment period underscoring the strategy’s capability to enhance therapeutic efficacy and safety. Notably, increasing time inside the target range, especially in the first days of treatment, has been linked to improved therapeutic outcomes (Borobia et al., 2009; Song et al., 2019; Yin et al., 2019).

There were limitations to this study, including the use of only trough concentration data, meaning the study needed to rely on literature data. Additionally, the lack of information on co-administered medications and genotypic data limited our ability to fully explain the observed interindividual variability. Also, safety concerns were not considered except for graft rejection, a problem that could be addressed through exposure-safety studies for the Hispanic population.

In conclusion, a popPK model of tacrolimus was developed for Hispanic renal transplant subjects using real-world data. Body weight and hematocrit explained variability in tacrolimus apparent clearance. The study highlighted an estimated 40% decrease in apparent clearance of tacrolimus in the Hispanic population compared to other populations with similar characteristics. Using the popPK model developed in the Hispanic population, an initial dose and an optimal dose titration strategy was proposed for tacrolimus. Based on model-based simulations, an initial dose of 0.1 mg/kg/day is proposed (similar to the tacrolimus label) for Hispanic patients receiving tacrolimus as part of their combination therapy with MMF (0.1 mg/kg/day), but increased caution is advised due to their decreased clearance and possible safety concerns. The proposed dose titration strategy demonstrated the potential to increase by 2.5-fold the proportion of patients achieving the therapeutic target (10–12 ng/mL) within the first week of treatment. These findings provide valuable insights into personalized dosing strategies and therapeutic drug monitoring in Hispanic patients receiving tacrolimus therapy.

## Data Availability

The data analyzed in this study is subject to the following licenses/restrictions: The collected data from the current study are not publicly available due to privacy and ethical restrictions but are available from the corresponding authors on reasonable request. Requests to access these datasets should be directed to MG, mgopalakrishnan@rx.umaryland.edu.

## Supplementary material

SUPPLEMENTARY FIGURE S1 Sensitivity analysis of dose selection on clearance estimation for sparse data.

SUPPLEMENTARY FIGURE S2 Comparative analysis of observed concentrations from White population and Hispanic population.
