# Supplementary material for: Optimizing tacrolimus dosing in Hispanic renal transplant patients: insights from real-world data
Source: Front Pharmacol. 2024 Sep 19;15:1443988. doi: 10.3389/fphar.2024.1443988 (PMC11446860; doi:10.3389/fphar.2024.1443988)
Supplement: Supplementary file 1 [file Table1.DOCX]

**Supplementary Table 1. Population pharmacokinetic model building steps for rich bioequivalence data**

| **Metric** | **-2LL** | **AIC** | **BIC** |
| --- | --- | --- | --- |
| First order 1 Compartment model | 2414.901 | 2428.901 | 2458.745 |
| First order 2 Compartment model | 2185.888 | 2209.888 | 2261.048 |
| First order 3 Compartment model | 2175.159 | 2207.159 | 2275.374 |
| Transit Absorption 1 Compartment model | 2256.578 | 2272.578 | 2306.685 |
| Transit Absorption 2 Compartment model | 2059.582 | 2081.582 | 2128.479 |
| Transit Absorption 3 Compartment model | 1768.308 | 1800.308 | 1868.522 |
| Zero Absorption 2 Compartment model | 2018.257 | 2042.257 | 2093.418 |
