# Supplementary figures and images for: Optimizing tacrolimus dosing in Hispanic renal transplant patients: insights from real-world data
Source: Front Pharmacol. 2024 Sep 19;15:1443988. doi: 10.3389/fphar.2024.1443988 (PMC11446860; doi:10.3389/fphar.2024.1443988)

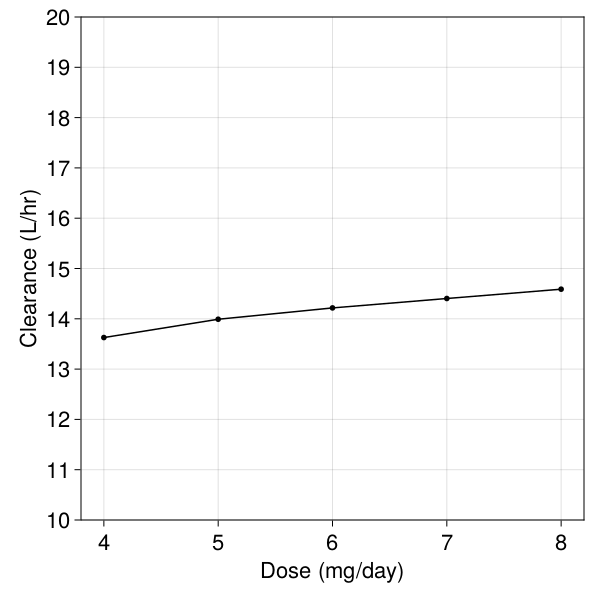

Supplement: Supplementary file 2 [file Image1.JPEG]

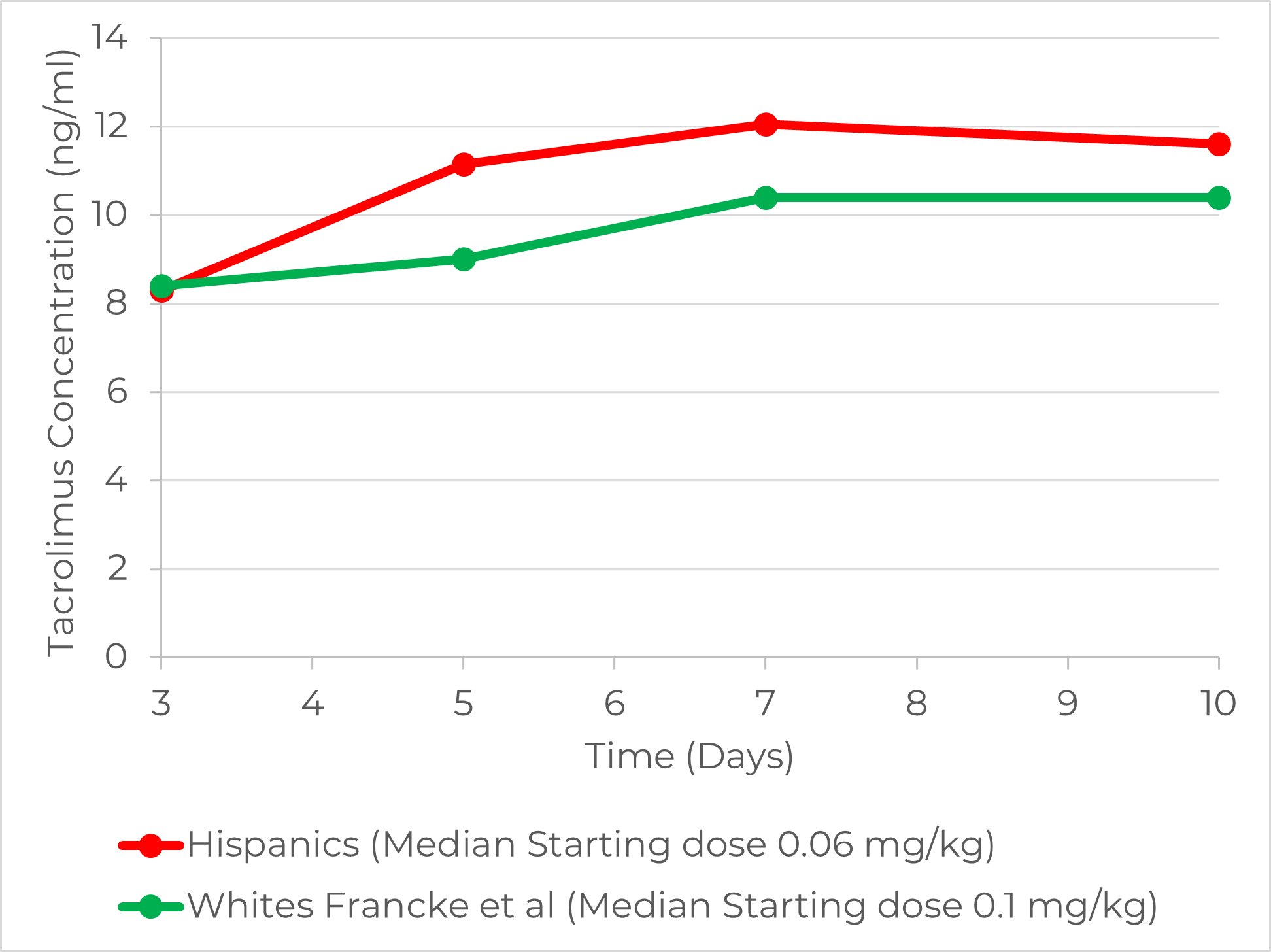

Supplement: Supplementary file 3 [file Image2.JPEG]
