# Supplementary material for: Optimizing tacrolimus dosing in Hispanic renal transplant patients: insights from real-world data
Source: Front Pharmacol. 2024 Sep 19;15:1443988. doi: 10.3389/fphar.2024.1443988 (PMC11446860; doi:10.3389/fphar.2024.1443988)
Supplement: Supplementary file 4 [file Table2.DOCX]

**Supplementary Table 2. Covariates impact on clearance parameter estimate.**

| **Covariate Influence** | **CL change compared to typical patient**  **(30% HCT, 70 kg)** |
| --- | --- |
| WT = 45 (kg) | 0.7-fold decrease |
| WT = 160 (kg) | 1.8-fold increase |
| HCT = 15(%) | 1.7-fold increase |
| HCT = 50(%) | 0.7-fold decrease |
